# Epidemiological Characteristics and Space-Time Analysis of the 2015 Dengue Outbreak in the Metropolitan Region of Tainan City, Taiwan

**DOI:** 10.3390/ijerph15030396

**Published:** 2018-02-26

**Authors:** Ting-Wu Chuang, Ka-Chon Ng, Thi Luong Nguyen, Luis Fernando Chaves

**Affiliations:** 1 Department of Molecular Parasitology and Tropical Diseases, School of Medicine, College of Medicine, Taipei Medical University, No. 250, Wuxing Street, Xinyi District, Taipei 11031, Taiwan; 2 College of Public Health, National Taiwan University, Taipei 10607, Taiwan; B507101064@tmu.edu.tw; 3 College of Medicine, Taipei Medical University, Taipei 11031, Taiwan; m142106004@tmu.edu.tw; 4 Instituto Costarricense de Investigación y Enseñanza en Nutrición y Salud (INCIENSA), Apartado Postal 4-2250, Tres Ríos, Cartago, Costa Rica; lfchavs@gmail.com; 5 Programa de Investigación en Enfermedades Tropicales (PIET), Escuela de Medicina Veterinaria, Universidad Nacional, Apartado Postal 304-3000, Heredia, Costa Rica

**Keywords:** dengue, space-time analysis, land use/land cover

## Abstract

The metropolitan region of Tainan City in southern Taiwan experienced a dengue outbreak in 2015. This manuscript describes basic epidemiological features of this outbreak and uses spatial and temporal analysis tools to understand the spread of dengue during the outbreak. The analysis found that, independently of gender, dengue incidence rate increased with age, and proportionally affected more males below the age of 40 years but females above the age of 40 years. A spatial scan statistic was applied to detect clusters of disease transmission. The scan statistic found that dengue spread in a north-south diffusion direction, which is across the North, West-Central and South districts of Tainan City. Spatial regression models were used to quantify factors associated with transmission. This analysis indicated that neighborhoods with high proportions of residential area (or low wetland cover) were associated with dengue transmission. However, these association patterns were non-linear. The findings presented here can help Taiwanese public health agencies to understand the fundamental epidemiological characteristics and diffusion patterns of the 2015 dengue outbreak in Tainan City. This type of information is fundamental for policy making to prevent future uncontrolled dengue outbreaks, given that results from this study suggest that control interventions should be emphasized in the North and West-Central districts of Tainan city, in areas with a moderate percentage of residential land cover.

## 1. Introduction

Dengue fever (DF) is caused by four distinct serotypes of dengue virus (DENV-I, DENV-II, DENV-III, and DENV-IV) (*Flavivirade*) which are transmitted by *Aedes aegypti* and *Aedes albopictus* [1]. DF has become the most important vector-borne disease in the tropical and sub-tropical regions, mainly circulating in Latin America, Southeast Asia, and South Asia [2]. More than 390 million cases are reported annually [1]. Severe dengue infection can be mediated through an antibody-dependent enhancement (ADE) mechanism which can induce dengue hemorrhagic fever or dengue shock syndrome [3]. The risk of severe dengue infection could be elevated if the secondary infection is caused by a different serotype of dengue virus.

The transmission risk of DF could be influenced by multiple environmental risk factors. Climatic conditions, including temperature, precipitation, and humidity, are the major drivers which have been highlighted in previous studies [4,5,6]. Higher temperatures could play a crucial role in shortening the duration of the mosquito’s life cycle or the extrinsic incubation period (EIP) of the virus, resulting in an increase of mosquito abundance and transmission probability as well [7]. An appropriate amount of precipitation is required to create more habitats for the mosquito vector [8,9]. Moreover, the increasing number of artificial containers produced by humans [10,11,12], alongside the requisite rainfall, also affords the *Aedes* spp. mosquito an increased survival [4,13]. 

In addition to climate drivers, socio-economic characteristics and landscape-level variables also have different impacts on dengue transmission [14]. A previous study conducted in Thailand indicated that irrigated fields or orchards near households could increase the transmission risk of dengue [15]. In Kenya, an irrigated area had higher dengue seroprevalence rates than areas without irrigation [16]. Population density played the most important role in the dengue transmission rate in Swat, Pakistan [17]. Those environmental factors affected the spatial and temporal distributions of both vector abundance and disease transmission [18,19]. Thus, it is very important to understand the spatial and temporal diffusion patterns of dengue transmission and its relevant environmental conditions as prevention resources can be deployed in the disease hotspots.

Dengue outbreaks have occurred in Taiwan every year since the early 1990s, and are usually ignited by imported cases of the virus from endemic countries [20]. The scale of an outbreak is determined by vector abundance and climate conditions in that year. Southern Taiwan, including Kaohsiung City and Tainan City, is a major dengue hotspot due to suitable climate conditions and high population density within the metropolitan regions. Earlier studies have focused on the dengue epidemics in Kaohsiung City because most cases have been reported from that area. Tainan City, considered to be the second largest dengue hotspot in southern Taiwan, experienced a dengue outbreak in 2015; however, studies relevant to this outbreak are still very rare. A previous study from southern Taiwan demonstrated that the El Niño Southern Oscillation (ENSO) index and local elevated temperature during spring and summer might be associated with the most recent outbreaks [21]. The spatial and temporal patterns of dengue transmission during this outbreak are seldom studied in detail. 

This study conducts a spatial epidemiological analysis of the 2015 dengue outbreak in the metropolitan region of Tainan City. There are three objectives in this study: (1) to explore the basic epidemiological features of the 2015 dengue outbreak in the metropolitan region of Tainan City; (2) to evaluate the occurrence of space-time clusters of the dengue outbreak; (3) to explore associations between specific land cover/land use (LCLU) types and the dengue incidence rate. The findings of this study could benefit public health agencies and workers in Tainan City in terms of future dengue outbreak control/prevention measures.

## 2. Materials and Methods

### 2.1. Study Area

**Figure 1:**
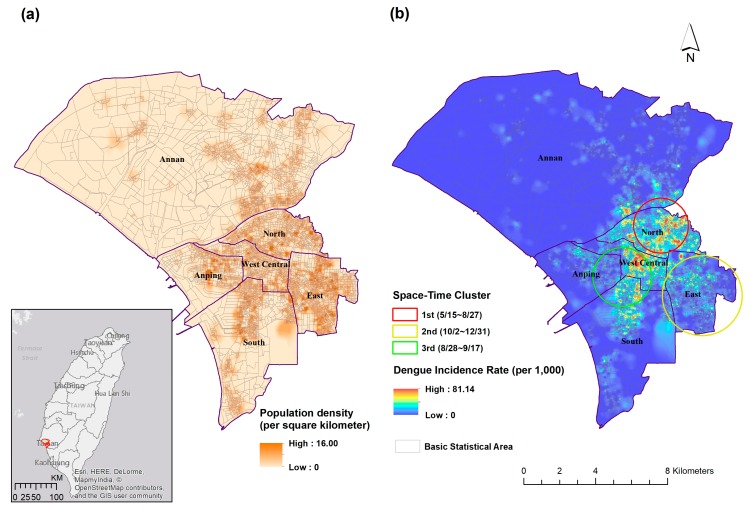
(**a**) The metropolitan region of Tainan City and population density; (**b**) Space-time cluster analysis of DF at BSA level in Tainan City metropolitan area, 2015.

This study focuses on the metropolitan area of Tainan City (22.98° N, 120.21° E), which experienced a dengue outbreak in 2015. Tainan City is located in the southwestern part of Taiwan where both *Ae. aegypti* and *Ae. albopictus* can be found. Tainan City is the sixth-largest city in Taiwan with a total population of 1.88 million, approximately 1 million of whom live within the metropolitan region (Figure 1a). The East district has the highest population density (14,049 per km^2^), followed by the North district (12,728 per km^2^), and West-Central district (12,356 per km^2^). The average temperature ranges from 17 to 29 °C and annual cumulative precipitation is approximately 1700 mm. The main rainy season is from June to August. The continuous accumulation of rainfall and high humidity are usually brought to this region by East Asia’s rainy season (plum rain) in May and June. Tainan City is a famous ancient cultural capital in Taiwan and the economic activities mainly comprise industry, agriculture, and tourism. Historically, Tainan City was the second largest dengue hotspot in southern Taiwan (Kaohsiung City was the major hotspot) due to its geographical location (tropical climate zone) and the activity of disease vectors.

### 2.2. Dengue Cases

DF has been classified as notifiable infectious disease category 2 in Taiwan. Physicians are required to report any suspected cases to the Taiwan Centers for Disease Control (CDC) within 24 h. A dengue case is confirmed by Taiwan CDC following the positive results of a serological test (IgM Enzyme-Linked Immunosorbent Assay), nucleotide sequence, or virus isolation following the standard protocol [22]. The nonstructural protein 1 (NS1) antigen detection has been employed as a method of rapid diagnosis since 2014. In 2015, the policy of releasing open data has been promoted by the Taiwanese government. Taiwan CDC has released dengue aggregated data at the basic statistical area (BSA) level. The administrative units of Taiwan are composed by a hierarchical structure, which can be assembled according to the 1st level dissemination area, 2nd level dissemination area, the 3rd level dissemination area (township), 4th level dissemination area (county), 5th level dissemination area (region), and 6th level dissemination area (nation). BSA is the finest administrative unit (the 1st level dissemination area) among them. Previous dengue studies in Taiwan have usually analyzed the data at the township or village level; however, the relatively coarse spatial resolution might have brought about some bias caused by the modifiable area unit problem (MAUP) [23,24,25]. Exploring the dengue incidence rate at the BSA level could minimize the impact of the MAUP. Moreover, patients’ privacy is protected because BSA data is aggregated. In this study, the spatial and temporal patterns of dengue incidence rates are analyzed at the BSA level. The LCLU analysis is applied at the 2nd level dissemination area (approximate village level) because a relatively large area size is required to calculate the percentages of different LCLU types. In 2015, a mere 17 imported cases were reported in Tainan City, so only locally acquired dengue cases were included in the analysis. The population data in the study area were obtained from Taiwan Ministry of the Interior for dengue incidence rate calculation. 

### 2.3. Land Cover/Land Use Types

LCLU data were acquired from the 2nd land use investigation which was launched by the National Land Surveying and Mapping Center, Ministry of the Interior, Taiwan (www.nlsc.gov.tw). A total of 103 LCLU types have been classified through integrating aerial photos, satellite images, and ground-based surveys. Six LCLU types were selected in the study according to two criteria: (1) the importance to vector ecology and dengue transmission and (2) availability of area size within the study unit. For instance, forest land cover is not included in the analysis because it is very rare within the metropolitan area of Tainan City. The percentages of the six LCLU types within the 2nd level dissemination area were calculated using ArcGIS 10.4 (ESRI, Redland, CA, USA) for further analysis. 

### 2.4. Statistical Analysis

The basic epidemiological characteristics, such as age-gender distributions of dengue incidence rates, were evaluated in the study area. Both the monthly and overall dengue incidence rates at BSA level were smoothed using the inverse distance weighted (IDW) interpolation technique to visualize the spatial distributions. The assumption of IDW is that the closer objects would have more similar characteristics. The method assigns greater weight to points around a nearby location, and the weights decrease as a function of distance [26]. The equation of IDW is set out below in Equation (1):

(1) $$Z\left(x\right)=\frac{\sum_{1}^{n}W_{i}Z_{i}}{\sum_{1}^{n}W_{i}}$$

where Z(x) is the estimated value, $W_{i}$ denotes the weighted value at location *i*, and $Z_{i}$ is the value at location *i*.

The spatial and temporal clusters of dengue in the Tainan metropolitan region were detected by spatial scan statistics. The space-time permutation model was adopted to evaluate the spatial and temporal hotspots simultaneously. The model is under the Poisson assumption with the formula listed in Equation (2):

(2) $${\left(\frac{c}{E\left[c\right]}\right)}^{c}{\left(\frac{C-c}{C-E\left[c\right]}\right)}^{C-c}I()$$

where *C* denotes the total number of cases and *c* is the observed number of cases within the window. E[c] is the expected number of cases within the window under the null-hypothesis. *I*() is an indicator function which is equal to 1 when the window has more cases than expected, and 0 otherwise [27]. The *p*-value is generated using the Monte Carlo hypothesis testing method with 999 times simulation, and the cut-off point is set at 0.05 [28]. This approach has been applied to dengue and other infectious diseases in our previous work [17,29]. The space-time permutation model was carried out by SaTScan V9.4 software (www.satscan.org, Boston, MA, USA).

The associations between dengue incidence and LCLU types were investigated using regression models. We compared the results using ordinary least squares (OLS) regression, the spatial error model, and the spatial lag model to check the impact of the spatial autocorrelation. In the spatial error models (3) and (4), the effect of autocorrelation is on error variance, where:

(3) y=Xβ+ε

(4) $$\varepsilon =\lambda W_{\varepsilon }+\varphi$$

We use y to denote the dengue incidence rate within the 2nd level dissemination area, *X* refers to different LCLU variables, β is regression coefficients. ε is a spatial error term which is composed of autoregressive coefficient (λ) and spatial weighted vector ($W_{\varepsilon }$). φ is the random error [30]. In the spatial lag model (5), the spatial autocorrelation is included as an autoregressive term ($W_{y}$) and ρ denotes the autoregressive coefficient [30]:

(5) $$y=\rho W_{y}+X\beta +\varepsilon$$

The model performance was evaluated by the adjusted R-squared (Adj R-squared) and Akaike information criterion (AIC) values [31]. The smallest AIC value indicates the best model [32,33]. The spatial regression models were carried out with GeoDa version 1.12. (Center for Spatial Data Science, Computation Institute, Chicago, IL, USA). All the map layouts were made with ArcGIS 10.4. The statistically significant variables in the final model were evaluated by the locally weighted scatterplot smoothing (LOWESS) approach [34] to check the intrinsic linearity with dengue incidence.

## 3. Results

### 3.1. Epidemiological Characteristics

**Figure 2:**
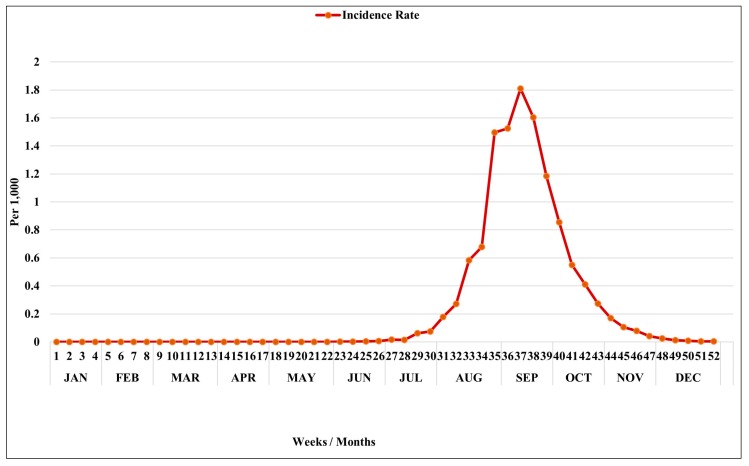
Weekly dengue incidence rate (per 1000) in Tainan City, 2015.

The total number of dengue cases in 2015 in Tainan City was 22,740 and the incidence rate was approximately 12.06 per 1000. The first autochthonous transmission of dengue case was reported in May 2015, but the number of cases began to increase in early July (week 27) (Figure 2). In August, the scale of the outbreak soared rapidly and reached its peak in the middle of September (week 38). The number of dengue cases dropped gradually after October (Figure 2). 

**Figure 3:**
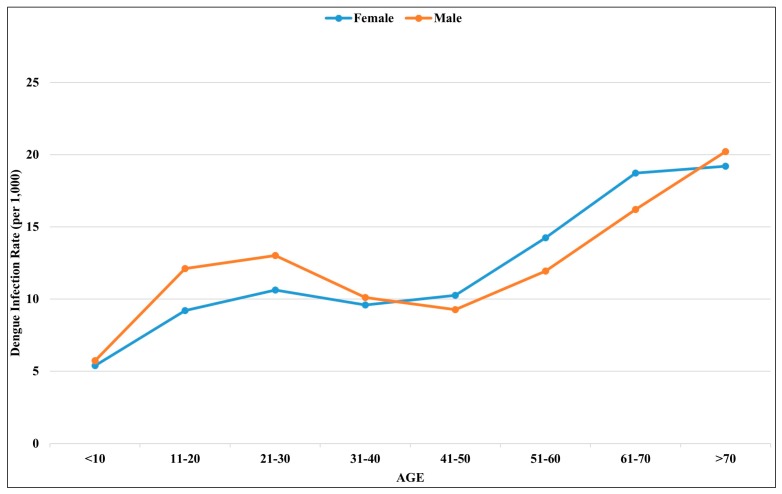
Age-gender distribution of dengue incidence rate (per 1000) in Tainan City, 2015.

Overall, the dengue infection rate increased with age with a sinusoidal shape pattern (Figure 3). The age–gender distribution revealed a higher dengue infection rate in females among the elderly population, especially for people over 40 years. Inversely, higher infection rates were observed among the male populations of children and young adults.

### 3.2. Spatial and Temporal Analysis

The spatial distribution of dengue in 2015 was significantly clustered in the North and West-Central districts (Figure 1b). The north-south direction from the North district to the South district can also be observed. Although the East district has the highest population density in Tainan City, the dengue incidence rate did not echo that of an area with a high susceptibility population. The space-time cluster statistics found three major clusters of dengue infection in the Tainan metropolitan area (Figure 1b). The major cluster was located in the North district in the early stage of the epidemic season (15 May–27 August). The second cluster was in the East district during the latter stage (2 October–31 December) and the third cluster covered the West-Central, South, and Anping districts between August and September (28 August–17 September). 

**Figure 4:**
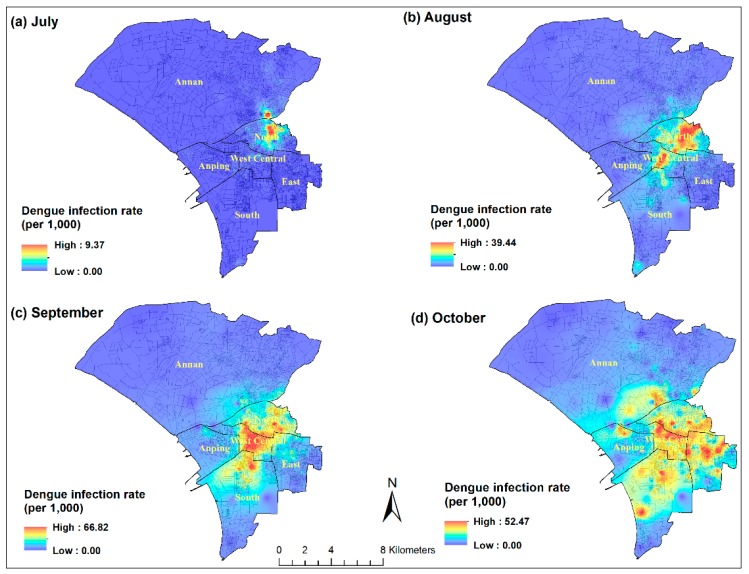
Monthly spatial distributions of dengue incidence rates (BSA level) in Tainan City during the outbreak season.

The month-specific spatial patterns also support the results from the space-time statistic (Figure 4). The outbreak originated in the North district by August and then the infected area expanded to the West-Central and South districts after August. In October, the wave of dengue transmission reached the surrounding region, including the most populated East district, and Anping and Annan districts.

### 3.3. LCLU Analysis

**Figure 5:**
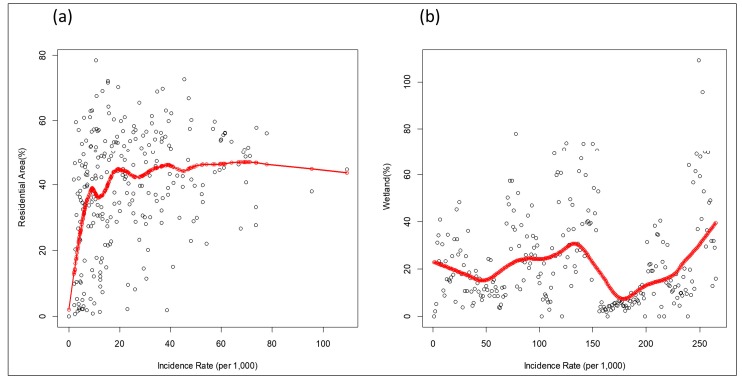
Non-linear relationships between dengue incidence rates (dots) and (**a**) residential area and (**b**) wetland, evaluated by LOWESS technique (red line).

The associations between LCLU types and dengue incidence rates were analyzed using different regression models. Overall, the residential area is the dominant LCLU type within the study unit (the 2nd level dissemination area), followed by business district, recreation area, and waterbody (Table S1; Figure S1). However, a high variability in the percentage of different LCLU types can also be observed. The variance inflation factor (VIF) of all the LCLU variables is small, which indicates that there is no strong multi-collinearity effect in the models. In the OLS model, residential area, business district, and agriculture were shown to have statistically significant effects on dengue incidence rates; however, the Moran’s I test indicated strong spatial autocorrelations (Moran’s I = 16.04, *p* < 0.001) (Table 1). Both the spatial lag regression model and spatial error model performed better than OLS after controlling for spatial effect (higher Adj R-squared and lower AIC). Residential area and wetland are the two statistically significant determinants associated with dengue infection but with different effects. If the spatial unit contains a higher percentage of residential area, a higher dengue incidence will occur. On the other hand, a higher percentage of wetland demonstrated a lower dengue risk. Since the residential area represented a significant risk factor, we investigated further to evaluate the linearity between the percentage of residential area and dengue incidence rate using the LOWESS approach. The result indicated the non-linear relationship between the two variables (Figure 5a). The positive linear pattern can be observed when the percentage of residential area increases from 0 to 40 percent; however, a higher percentage of residential area does not show a higher transmission risk of dengue. Non-linearity was also detected between wetland and dengue infection (Figure 5b). Although a higher dengue incidence rate related to a lower percentage of wetland, the positive association can be observed in the extremely high infection rate. 

## 4. Discussion

The global burden of DF has rapidly increased since 1990, and many countries in Southeast Asia (including Taiwan) have experienced annual outbreaks [2,17,21,35,36]. Most previous studies of dengue outbreaks in Taiwan have focused on the major epidemic center in Kaohsiung City [37,38,39]. This is the first study to evaluate the epidemiological characteristics and spatial-temporal patterns of dengue incidence rates in Tainan City. A fundamental difference in the age and age-gender distribution trends for dengue transmission was observed. In most dengue endemic countries in Southeast Asian and Latin American, children are typically considered the most vulnerable group [40,41,42]. Guo et al. analyzed dengue outbreaks in Guangdong (China) from 2005–2011, and found that the highest incidence rate was observed in the young adult population (20–30 years old) [43]. Similar to the patterns in Taiwan, dengue outbreaks in Singapore also exhibit an increasing incidence with age [44]. The different epidemiological characteristics might be related to several environmental determinants, economic development statuses, and interactions between humans and vectors. The higher dengue infection rate among the elderly population in Tainan City could raise a critical issue related to comorbidities. Dengue infection with other chronic conditions, like cardiovascular disease or diabetes, could exacerbate the progression of DF and increase the mortality rate [45,46,47]. Thus, disease prevention work should be strengthened among such vulnerable populations.

To our knowledge, this is the first time a report on the reverse pattern of age–gender distributions of dengue infection has been presented. Two hypotheses could be derived from such an observation. First, the transmission risk, which is mediated through the environmental or behavioral characteristics, might be higher in the senior female population and cause higher infection rates in the study area. The other hypothesis might be associated with physiological or immunological responses. Older females might demonstrate a higher symptomatic infection rate than elderly males, so they have a higher chance of being reported by the physicians. Both of these hypotheses are worthy of further investigation. 

The spatial and temporal pattern of the dengue outbreak in Tainan City shows a clear north-south direction. The outbreak originated in the North district and then spread to the West-Central and South districts. The major hotspot in September was clustered within the West-Central district, which is the oldest community with many ancient buildings and a high population density in the city. On the other hand, the most populated East district is a relatively newly developed area where the higher dengue incidence rate occurred in the later stage (October) of the outbreak. The risk maps highlight the important areas where public health workers should enhance control efforts in the future. 

The spatial and temporal analysis of DF highlights the significant association with population density, which is also linked to the higher percentage of residential area derived from the results of the spatial regression model. The association between dengue and urbanized characteristics or population density has been mentioned in other studies [48,49,50]. One study, which applied the boosted regression trees approach, indicated that human settlements and waterbodies are major risk factors for dengue cases in Malaysia [51]. The link is mediated through vector abundance and contact probability of the human host. Our study further demonstrates that the association is non-linear. A moderate percentage (about 40%) of the residential area has a stronger association with a higher dengue incidence rate. One study has also shown such a non-linear pattern between dengue outbreak and population density in the Pearl River Delta, China [52]. The non-linear association might be attributed to the complicated interactions between vector activity and human behaviors, which include people movements, personal protective awareness, or response to the health education, in a finer environmental setting. Vector control also played an important role to control the scale of the outbreak. The percentage of wetland also exhibited the non-linear pattern with dengue incidence; however, the wetland is mainly in the suburban or coastal region within the Annan and Anping districts where the dengue incidence rate is relatively lower than the city center. 

The outbreak in Tainan could have been triggered by the unusual climate factors which have been proved by our previous work [21]. With the increasing effects of climate change, similar outbreaks could occur again in the future. Our study provides a preliminary result showing dengue diffusion patterns in space and time. Public health workers can focus on the potential hotspots in order to strengthen disease/vector control measures. The vulnerable population can also take more preventive activities through community education to reduce the risk of infection. 

The limitation in the study is that regular vector surveillance data is not available in the study region. The impact of different environmental determinants on the vector population is therefore unknown. There is also no way to analyze the spatial and temporal relationships between dengue incidence and vector population dynamics. Routine vector surveillance systems can help public health workers to monitor vector abundance in a longitudinal way, which more accurately identifies the level of dengue transmission risk [53]. The Taiwan CDC and the national health research institutes have started to develop an intelligent mosquito trap, which might provide the opportunity for establishing a vector surveillance system in the future. 

## 5. Conclusions

This is the first study to describe the fundamental epidemiological characteristics of a dengue outbreak in 2015 in Tainan City, Taiwan. The dengue infection rate increased with age, and the female population exhibited a higher infection rate if they were older than 40 years. The spatial and temporal analysis indicated that the DF spread from north to south in the early stage and then expanded into the surrounding area in the later stage. A non-linear association between the percentage of residential area in a district and the dengue incidence rate was highlighted in the spatial regression model. Our findings provide a preliminary analysis which may support public health workers to focus disease control efforts in dengue hotspots of Tainan City. The study also indicated that dengue transmission risk could evolve with urban development. High risk population might be shifted in space, time, socio-economic status, and environmental conditions. The dengue surveillance system should be strengthened to confront the increasing likelihood of future dengue outbreaks.

## Figures and Tables

**Table 1:** Spatial regression analysis of DF and LCLU types in the metropolitan region of Tainan City, 2015.

Variables	OLS		Spatial Lag Model		Spatial Error Model	
	Coefficient	*p*-Value	Coefficient	*p*-Value	Coefficient	*p*-Value
Residential Area	0.23	0.002	0.09	0.03	0.08	0.04
Recreation Area	0.12	0.55	−0.12	0.31	−0.11	0.28
Business Area	1.19	<0.001	0.25	0.12	0.14	0.45
Agriculture	−0.31	0.02	−0.05	0.55	−0.04	0.67
Wetland	−0.29	0.35	−0.41	0.02	−0.54	0.001
Water	−0.04	0.87	−0.12	0.48	−0.19	0.34
Moran’s I test	16.04	<0.001	-	-	-	-
AIC	2287		2049.06		2051.33	
Adj R-squared	0.21		0.74		0.73	

OLS: ordinary least squares; AIC: Akaike information criterion; Adj R-squared: adjusted R-squared.

## Supplementary Materials

The following are available online at http://www.mdpi.com/1660-4601/15/3/396/s1, Figure S1: LCLU percentages within the 2nd level dissemination area in the metropolitan region of Tainan City, Table S1: The selected LCLU types in the analysis.
