# Supplementary material for: Epidemiological Characteristics and Space-Time Analysis of the 2015 Dengue Outbreak in the Metropolitan Region of Tainan City, Taiwan
Source: Int J Environ Res Public Health. 2018 Feb 26;15(3):396. doi: 10.3390/ijerph15030396 (PMC5876941; doi:10.3390/ijerph15030396)
Supplement: Supplementary file 1 [file ijerph-15-00396-s001.pdf]

Supplementary Materials

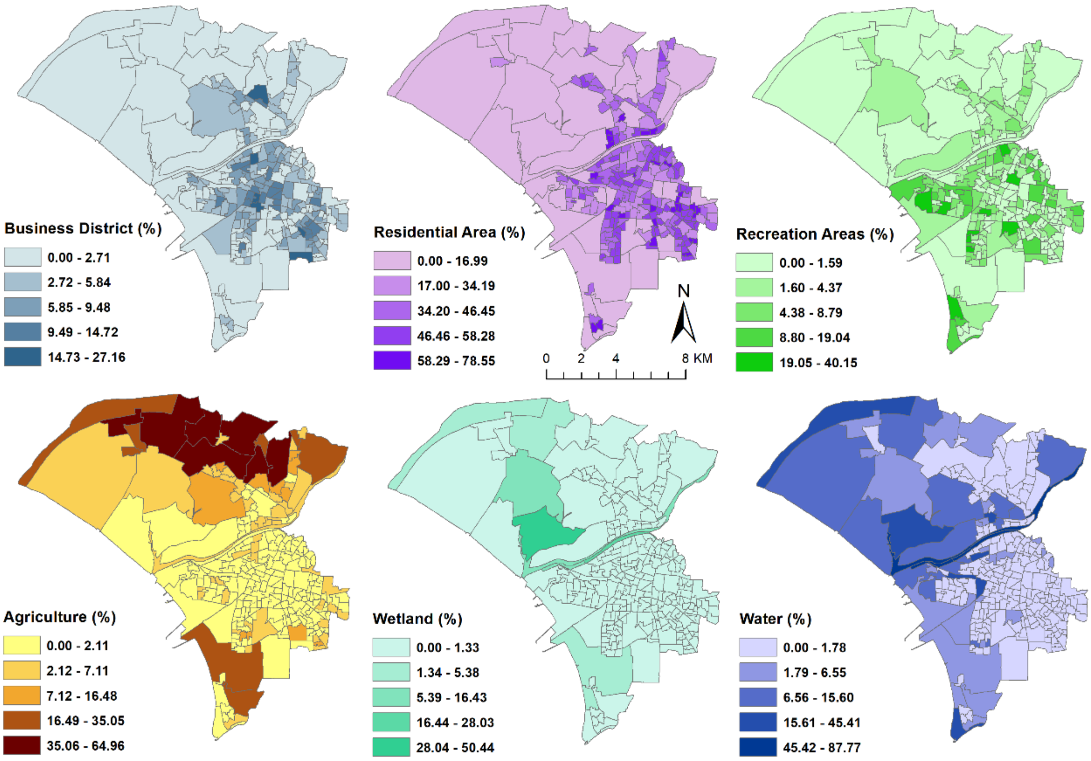

Figure S1. LCLU percentages within the 2nd level dissemination area in the metropolitan region of Tainan City.

Table S1. The selected LCLU types in the analysis

LCLU Types	Mean (%)[§]	Maximum (%)	Minimum (%)	VIF*
Business district	4.46	27.16	0	1.14
Agriculture[#]	0.78	31.03	0	1.47
Residential area	37.83	78.55	0	1.97
Recreation area	3.85	40.15	0	1.25
Wetland	0.58	50.44	0	1.45
Water	2.97	87.77	0	1.65

§: The mean/ maximum/ minimum percentages of LCLU within the 2nd level dissemination area in the metropolitan region of Tainan City.

Agriculture includes rice farms, orchards, and drought-tolerant crops

* variance inflation factor
